# An inventory of European data sources to support pharmacoepidemiologic research on neurodevelopmental outcomes in children following medication exposure in pregnancy: A contribution from the ConcePTION project

**DOI:** 10.1371/journal.pone.0275979

**Published:** 2022-10-14

**Authors:** Joanne Given, Rebecca L. Bromley, Florence Coste, Sandra Lopez-Leon, Maria Loane

**Affiliations:** 1 Faculty of Life and Health Sciences, Ulster University, Coleraine, Northern Ireland, United Kingdom; 2 Division of Neuroscience and Experimental Psychology, University of Manchester, Manchester, United Kingdom; 3 Royal Manchester Children’s Hospital, Manchester Academic Health Science Centre, Manchester, United Kingdom; 4 Sanofi Aventis R&D, Chilly Mazarin, France; 5 Novartis Pharmaceuticals Corporation, One Health Plaza, East Hanover, NJ, United States of America; 6 Center for Pharmacoepidemiology and Treatment Science, Rutgers University, New Brunswick, NJ, United States of America; Norwegian Institute of Public Health: Folkehelseinstituttet, NORWAY

## Abstract

**Background:**

Studies on medication safety in pregnancy are increasingly focusing on child neurodevelopmental outcomes. Establishing neurodevelopmental safety is complex due to the range of neurodevelopmental outcomes and the length of follow-up needed for accurate assessment. The aim of this study was to provide an inventory of European data sources for use in pharmacoepidemiologic studies investigating neurodevelopment following maternal medication exposure.

**Method:**

The EUROmediSAFE inventory of data sources in Europe for evaluating perinatal and long-term childhood risks associated with in-utero exposure to medication was updated by contacting colleagues across 31 European countries, literature review and internet searches. Included data sources must record at least one neurodevelopmental outcome and maternal medication use in pregnancy must be available, either in the data source itself or through linkage with another data source. Information on the domain of neurodevelopment, measure/scale used and the approach to measurement were recorded for each data source.

**Results:**

Ninety data sources were identified across 14 countries. The majority (63.3%) were created for health surveillance and research with the remaining serving administrative purposes (21.1% healthcare databases,15.6% other administrative databases). Five domains of neurodevelopment were identified—infant development (36 data sources,13 countries), child behaviour (27 data sources, 10 countries), cognition (29 data sources, 12 countries), educational achievement (20 data sources, 7 countries), and diagnostic codes for neurodevelopmental disorders (42 data sources, 11 countries). Thirty-nine data sources, in 12 countries, had information on more than one domain of neurodevelopment.

**Conclusion:**

This inventory is invaluable to future studies planning to investigate the neurodevelopmental impact of medication exposures during pregnancy. Caution must be used when combining varied approaches to neurodevelopment outcome measurement, the age of children in the data source, and the sensitivity and specificity of the outcome measure selected should be borne in mind.

## Introduction

Studies on drug utilisation in pregnancy report that up to 70–90% of women use one or more medications during pregnancy [1, 2]. Despite this, only 5% of all medications have been tested for use in pregnancy and appropriate safety information recorded on the medication patient information leaflet [3]. A review of drugs assessed by the Food and Drug Administration (FDA) reported that 97.7% of the drugs were classified as having an “undetermined” teratogenic risk in human pregnancy, and the mean time to determine a risk was 27 years [4]. There is therefore an urgent need for knowledge of medication use and safety during pregnancy.

Historically research has concentrated on congenital anomalies but there is increasing interest in the potential for medication exposure during pregnancy to adversely impact neurodevelopment (ND) [5, 6]. The term ND covers a diverse range of brain functions including intellectual abilities, language, attention, and cognition, but also encompasses motor development, social skills, behavioural and emotional regulation. Such diversity means that there are many outcomes which fall within the category of ND and even more numerous ways to define and measure functioning in these skill areas. As different cognitive, motor, and social skill sets mature at different ages certain effects will only become evident as age relevant skills emerge and mature. For example, the expected complexity of social skills as a two-year-old is far less than the complex abilities in both verbal and non-verbal social communication expected in the adolescent years. As different domains of ND may be differentially impacted upon by teratogen exposure a wide variety of outcomes must therefore be assessed at appropriate ages to establish ND safety [5].

It is a priority to increase efforts to detect medications which convey risk to the developing child’s brain. This is a particular concern for medications, which affect the central nervous system and which can cross the placental barrier [7–12], such as the antiseizure medications (ASMs), antidepressants and antipsychotics. For example, exposure to the ASM valproate during pregnancy has been associated with reduced IQ scores, particularly verbal IQ, attention deficit hyperactivity disorder (ADHD) and Autism spectrum disorder (ASD) [13–15]. Isotretinoin exposure in utero has been found to reduce IQ scores, but had a more significant impact on visual-spatial skills [16]. Finally, there is conflicting evidence regarding the risk of ASD [17–21] following in utero exposure to selective serotonin reuptake inhibitor (SSRI) antidepressants. Recent investigations using detailed language assessments of every exposed child in the cohort raise the possibility that the primary deficit may be in the language domain and in particular, pragmatic language [22]. There is therefore a clear risk of lifelong ND impairments associated with certain medication exposures and efforts should be made to detect those which carry this risk as soon as possible.

To date most evidence relating to the impact of medication exposure on ND has derived from observational studies and population-based cohort studies utilising electronic records. Both have inherent methodological limitations and strengths. Traditional observational cohort studies recruit pregnant women directly within hospital or community-based health care settings and the participants are followed up using study specific standardised protocols, often utilising direct blinded assessment of the child through the postnatal years, with good control over confounding variables. However, such methodologies may have lower statistical power, have relatively short follow-up periods (typically only up to pre-school age) and can be financially costly. Cohorts derived from population based electronic records alternatively, offer large numbers of exposed children often across a broader range of maternal indications. However, these are often based on diagnostic codes recorded or service referrals [23], data comes from multiple assessors who are not blinded to the medication exposure history of the child and often have more limited information on potential confounding variables (e.g. wider family history of disorders, parental intellectual level etc). Thus, pharmacoepidemiology research in relation to ND will require a combination of methodological approaches which cover a range of outcomes, with investigations extending into the adolescent years.

The growth of secondary data sources, with mother-baby linkages and large population sizes, raises the potential for timely evaluations of neurodevelopmental safety following maternal medication use during pregnancy. The aim of this study was to provide an inventory of European data sources with the potential to be used in pharmacoepidemiologic studies investigating ND in relation to maternal medication exposure. The objectives of the study were to capture how ND outcomes are recorded within these data sources and to consider their strengths and limitations for assessing ND outcomes.

## Material and methods

The Innovative Medicines Initiative ConcePTION Project is a large collaborative project between academic, regulatory and industry partners [24], with the primary aim to create a system of improved monitoring and communicating safety of medicines use in pregnancy and breastfeeding. One of the tasks of the ConcePTION project was to “identify data sources that can be used for medication utilisation and medication safety studies”. An inventory of available data sources in 28 EU Member States for evaluating perinatal and long-term childhood risks associated with in-utero exposure to medication was published by the EUROmediSAFE consortium in 2018 [25]. The full EUROmediSAFE inventory is available at http://www.euromedicat.eu/content/EUROmediSAFE Inventory_Finalv2_2018_07_06.pdf. We reviewed, updated and extended the EUROmediSAFE inventory to provide the ConcePTION Consortium and other beneficiaries with a complete inventory of European data sources which could be considered for medication utilization and medication safety studies in pregnancy available at https://www.imi-conception.eu/wp-content/uploads/2019/09/ConcePTION_D1.1_spreadsheet-containing-all-additional-data-sources-for-the-ConcePTION-Data-Source-Catalogue.pdf. This article relates specifically to the identification of data sources which could be used for investigations of longer-term ND outcomes in pharmacoepidemiologic studies.

### Identification of data sources

A number of different methods were used to identify potential sources of information. First, we contacted our colleagues in the EUROCAT (European surveillance of congenital anomalies) network /EUROmediCAT (European congenital anomalies and medication safety) consortium with members in 21 countries and Euro-Peristat (European surveillance of perinatal health) with members in 31 countries. The purpose of the study was explained and they were invited to review the contents of the EUROmediSAFE inventory and to provide updates or add new electronic or linkable data sources that could potentially be useful for studies on medication use and safety in pregnancy in their country. They were asked to specifically consider data sources for capturing ND outcomes. This work was supplemented by a workshop held at a Euro-Peristat meeting in 2019 which was attended by approximately 50 Euro-Peristat members from across Europe and where we presented our findings and sought to identify additional sources in the countries that had not responded to our email requests.

Secondly, we conducted a literature review using the Embase database to identify data sources with outcomes following SSRI exposure during pregnancy in July 2019, which was updated in July 2020. SSRIs were used to identify data sources as the authors are conducting pharmacoepidemiologic studies on SSRI exposures in pregnancy, but other medications such as antiepileptic drugs could equally have been used. The search terms used are included in S1 File. The search was limited to the English language and publications within the last 10 years. Conference abstracts were excluded. Articles exploring ND outcomes were identified based on the title and the abstract and their sources checked against those already identified for the inventory.

The literature review was further supplemented by searches of national statistical organisation websites (for e.g. Statistics Denmark, Statistics Norway) and the https://www.birthcohorts.net/ website was searched to identify any missing birth cohorts with maternal medication exposure in pregnancy and ND outcomes [26].

### Eligibility for inclusion of data sources in this inventory

- Secondary source of electronic data with potential to be linked (i.e. primary purpose of collection was not for medication exposure investigations)
- Information on at least one ND outcome (e.g. behaviour, cognition, emotional regulation outcomes) using diagnostic codes, questionnaires, medical charts etc.
- Information regarding maternal medication use in pregnancy, either in the data source itself or through linkage with another data source.

### Exclusion criteria

Prospective Studies such as case reports, clinical studies, randomised controlled trials and adverse drug reaction databases were excluded as these have small sample sizes or selected populations.

### Classification of data sources

Data sources were classified according to the type of data available, see S2 File for more information:

Healthcare databases:

- Hospital (Admission/Episode/Discharge) databases
- Primary care databases
- Administrative health insurance claims databases
- Child surveillance databases

Other administrative databases for the delivery of services, reimbursement of costs:

- Educational databases
- Register of disability

Health surveillance and research databases:

- Disease registries
- Birth cohorts
- Research Cohort by Data Linkage

### Domain of ND

Information on the domain of ND, categorised based on the type of data collected, the measure/scale used (e.g. psychometric questionnaires, diagnostic codes) and the approach to measurement (e.g. parent completed questionnaire, clinician judgement) were recorded for each data source. This information was extracted by authors JG and RB from information publicly available relating to the data source such as a website or publications.

Ethical approval was not required for this study.

## Results

Fifty data sources with ND outcomes were listed in the EUROmediSAFE inventory and contacts in EUROCAT/EUROmediCAT and Euro-Peristat identified an additional 27 data sources. The literature search resulted in 2,798 citations. Based on manual review of the abstract, articles were excluded when related to pre-clinical, genetic, epigenetic, case reports, case series, where the outcome was out of scope (e.g. child’s depression; imaging, post-partum depression, pulmonary hypertension), or the exposures / intervention were not SSRIs (other drugs, stress, smoking). Thirteen data sources were identified in the literature (8 of these had already been identified in the EUROmediSAFE inventory or by contacts). An additional 8 data sources were identified by web searches.

**Fig 1:**
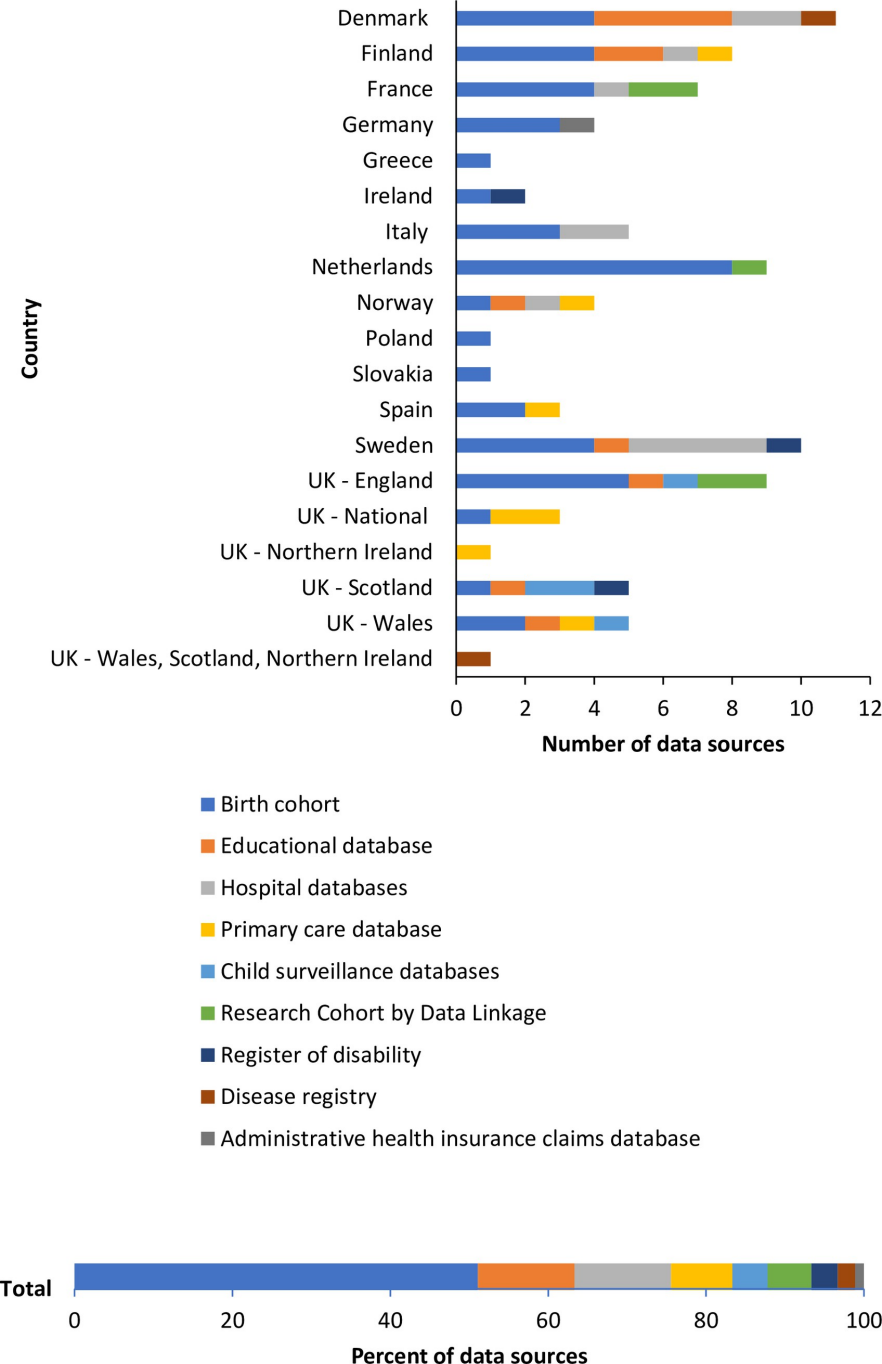
Type of data source identified across each country and percent of all data sources by type.

In total 90 data sources were identified across 14 countries. The majority (63.3%) were created for health surveillance and research with the remaining serving administrative purposes (21.1% healthcare databases and 15.6% other administrative databases). As can be seen in Fig 1, half of the data sources were birth cohorts. While the other types of data source were less numerous, these generally included much larger populations than the birth cohorts, for some the entire population of a country, and so represented a much larger sample.

Across the data sources identified the ND outcomes available were categorised, based on the type of data collected, into five domains of ND—infant development, child behaviour, cognition, educational achievement, and the presence of diagnostic codes for neurodevelopmental disorders. There is inevitable overlap between certain categories but this classification system allows users to select data sources by area of ND which may be relevant to their investigations. In 39 data sources, across 12 countries, it is possible to examine more than one domain of ND.

Information on infant development was available in 36 data sources across 13 countries, see Table 1, and was recorded in all types of databases except for the education and health insurance claims databases. Assessment of infant development varied, based on clinician judgement/routine health care, direct/objective assessment and/or parental completed questionnaires. In the birth cohorts’ bespoke questionnaires and a wide range of recognised measures, such as the Ages and Stages Questionnaire, Bayley Scales of Infant Development and Denver Developmental Screening Test were used to assess infant development. Such measures were also available in some of the non-cohort data sources such as the child surveillance and disease registries. Assessments made as part of routine healthcare were the predominant source of information in the other types of data source. Here infant development was assessed in routine health and developmental evaluations, service use records, READ (a coding system used in UK primary care) and ICD-10 (International Classification of Disease 10th edition) diagnosis codes, health visitor records and records of referral for support relating to developmental delay.

**Table 1:** Data sources which record infant development.

Country	Geographic coverage	Database name	Sub-type of data	Approach to Measurement	Measure/Scale Used^
**Denmark**	Births at Skejby Hospital, Denmark	Aarhus Birth Cohort	Birth cohort	Parent complete questionnaire/report	ASQ
**Denmark**	Copenhagen County	Copenhagen Child Cohort 2000 (CCC2000)	Birth cohort	Direct/Objective Assessment	BSID-II (1.5 years)
				Clinician judgement/routine health care	Health visitor records
**Denmark**	National	Danish National Birth Cohort	Birth cohort	Parent complete questionnaire/report	Bespoke Questionnaire; Developmental Coordination Disorder Questionnaire
**Finland**	Northern Finland	Northern Finland birth cohort of 1966	Birth cohort	Parent complete questionnaire/report	Bespoke Questionnaire
**France**	Haute-Garonne (south-west France)	EFEMERIS (Evaluation in Pregnant Women of MEdicaments and their RISK)	Research Cohort by Data Linkage	Clinician judgement/routine health care	Bespoke Questionnaire
**France**	National	EPIPAGE 2 Cohort Study	Birth cohort	Parent complete questionnaire/report	ASQ
				Clinician judgement/routine health care	GMFCS; SCPE diagnostic criteria
**France**	National	Etude Longitudinale Francaise depuis l’Enfance (ELFE)	Birth cohort	Parent complete questionnaire/report	CDI
**France**	Haute-Garonne (south-west France)	POMME (PrescriptiOn Médicaments Mères Enfants)	Research Cohort by Data Linkage	Clinician judgement/routine health care	Routine examination
**Germany**	Leipzig	LIFE Child	Birth cohort	Direct/Objective Assessment	BSID-III
**Greece**	Crete	Mother Child Cohort in Crete (RHEA)	Birth cohort	Direct/Objective Assessment	BSID-III
**Ireland**	County Cork	BASELINE: Babies after SCOPE	Birth cohort	Direct/Objective Assessment	BSID
**Italy**	National	Nascita e INFanzia: gli Effetti dell’Ambiente (NINFEA)	Birth cohort	Parent complete questionnaire/report	Bespoke questionnaire
**Italy**	Florence, Rome, Trieste, Turin and Viareggio	Piccolipiù	Birth cohort	Unclear	Unclear
**Italy**	Emilia Romagna Region	SINPIA ER-Flusso informativo per i servizi di neuropsichiatria infantile dell’infanzia e dell’adolescenza dell’Emilia Romagna	Hospital database*	Clinician judgement/routine health care	ICD-10; service use records
**Netherlands**	Rotterdam	Generation R	Birth cohort	Direct/Objective Assessment	CDI; MB-CDI Short Form
**Netherlands**	Rotterdam	Generation R Next	Birth cohort	Direct/Objective Assessment	Eye-tracking
**Netherlands**	Westelijke Mijnstreek region	LucKi Birth Cohort Study	Birth cohort	Direct/Objective Assessment	Van Wiechen classification of psychomotor development; Unknown language assessment
**Netherlands**	National	PRIDE Study (PRIDE: PRegnancy and Infant DEvelopment)	Birth cohort	Parent complete questionnaire/report	ASQ
**Netherlands**	five municipalities in the North of The Netherlands	Tracking Adolescents’ Individual Lives Survey (TRAILS) NEXT	Birth cohort	Direct/Objective Assessment	Bespoke observation and tasks
				Parent complete questionnaire/report	Bespoke questionnaire
**Norway**	National	Norwegian Mother, Father and Child Cohort Study (MoBa)	Birth cohort	Parent complete questionnaire/report	ASQ; Dale sentence complexity task; NVCC; SCQ
**Poland**	Eight regions of Poland	REPRO_PL Polish Mother and Child Cohort Study	Birth cohort	Direct/Objective Assessment	BSID-III
**Slovakia**	Eastern Slovakia: Michalovce	Slovak PCB study Exposure to polychlorinated biphenyl	Birth cohort	Direct/Objective Assessment	BSID-II
**Spain**	Seven Spanish regions (Ribera d’Ebre, Menorca, Granada, Valencia, Sabadell, Asturias, and Gipuzkoa)	INMA-Environment and Childhood Project (INMA Project)	Birth cohort	Direct/Objective Assessment	BSID; Dubowitz Developmental Screening Test
**UK—England**	Avon, England	ALSPAC-G2 (second generation of The Avon Longitudinal Study of Parents and Children)	Birth cohort	Parent complete questionnaire/report	Bespoke developmental questionnaire
**UK—England**	Avon, England	Avon Longitudinal Study of Parents & Children/Children of the 90s (ALSPAC)	Birth cohort	Parent complete questionnaire/report	Denver Developmental Screening Test
**UK—England**	Bradford, England	Born in Bradford/Born in Bradford Growing up	Birth cohort	Direct/Objective Assessment	CKAT
**UK—England**	England	Community Services Data Set (CSDS)	Child surveillance databases	Parent complete questionnaire/report	ASQ
**UK—England**	Southampton	Southampton Women’s Survey	Birth cohort	Direct/Objective Assessment	WPPSI; CANTAB
**UK—England**	Wirral, England	Wirral Child Health and Development Study	Birth cohort	Direct/Objective Assessment	BSID-III; NBAS; LabTAB; Physiological responses
**UK—Northern Ireland**	Northern Ireland	General Practitioner Information Platform	Primary care database	Clinician judgement/routine health care	Read codes
**UK—Scotland**	Scotland	Child Health Systems Programme—Pre-School (CHSP Pre-School)	Child surveillance databases	Parent complete questionnaire/report	ASQ; PEDS; PEDS:DM; SOGS II; SSLM. For subset: M-CHAT, PEDS, PEDS:DM, SOGS II, SSLM, Eyberg Child Behaviour Inventory,
**UK—Scotland**	Scotland	Growing up in Scotland (GUS)	Birth cohort	Parent complete questionnaire/report	Bespoke milestone questionnaire
**UK—Scotland**	Scotland	Support Needs System (SNS)	Register of disability	Clinician judgement/routine health care	Referrals
**UK—Wales**	Swansea, Wales	Growing up in Wales	Birth cohort	Parent complete questionnaire/report	Bespoke questionnaire
**UK—Wales**	Wales	National Community Child Health Database	Child surveillance databases	Clinician judgement/routine health care	Routine health and developmental evaluations
**UK—Wales, Scotland, Northern Ireland**	Wales, Scotland, Northern Ireland	The National Neonatal Research database (NNRD)	Disease registry	Parent complete questionnaire/report	Bespoke questionnaire
				Direct/Objective Assessment	BSID-III; Griffiths Test; SGS

* Admission, Episode, Discharge

^See S3 File for abbreviations of ND measurement tools

Assessments of child behaviour were available in 27 data sources across 10 countries, recorded in birth cohorts and child surveillance databases, see Table 2. Behaviour was based on child self-report, direct/objective assessment, parent completed questionnaire/report and teacher review/routine education. Behaviour was assessed using a variety of measurements/scales such as the Strengths & Difficulties Questionnaire, Child Behavior Checklist, Child Behavior Questionnaire, and bespoke questionnaires.

**Table 2:** Data sources which record child behaviour.

Country	Geographic coverage	Database name	Sub-type of data	Approach to Measurement	Measure/Scale Used^
**Denmark**	Copenhagen County	Copenhagen Child Cohort 2000 (CCC2000)	Birth cohort	Parent complete questionnaire/report	CBCL/1.5–5, SDQ, ITSCL
**Denmark**	National	Danish National Birth Cohort	Birth cohort	Child Self-Report	SDQ
				Parent complete questionnaire/report	SDQ
				Teacher Review/Routine Education	SDQ
**Denmark**	Municipality of Odense	Odense Child Cohort	Birth cohort	Parent complete questionnaire/report	CBCL/1.5–5; CBCL/6-18, SRS
**Finland**	Southwest Finland Hospital District and the Åland Islands	FinnBrain Birth Cohort Study (FinnBrain)	Birth cohort	Parent complete questionnaire/report	IBQ-R; ECBQ-R
				Direct/Objective Assessment	Lab-TAB
**Finland**	Helsinki	Perinatal Adverse events and Special Trends in Cognitive Trajectory (PLASTICITY)	Birth cohort	Parent complete questionnaire/report	CBCL
				Child Self-Report	CBCL-YSR, BS
**Finland**		Prediction and Prevention of Preeclampsia and Intrauterine Growth Restriction (PREDO)	Birth cohort	Parent complete questionnaire/report	CBCL/1.5–5
**France**	Nancy and Poitiers	EDEN—Study on the pre and early postnatal determinants of child health and development	Birth cohort	Parent complete questionnaire/report	SDQ; EAS
**France**	Brittany	PELAGIE study (Endocrine Disruptors: Longitudinal Study on Anomalies in Pregnancy, Infertility and Childhood)	Birth cohort	Parent complete questionnaire/report	SDQ
**Germany**	Munich, Leipzig, Wesel, and Bad Honnef, Germany	Influence of life-style factors on the development of the immune system and allergies in East and West Germany (LISA PLUS)	Birth cohort	Parent complete questionnaire/report	SDQ
**Germany**	Leipzig	LIFE Child	Birth cohort	Parent complete questionnaire/report	SDQ (10–18); Bespoke Hyperkinetic Questionnaire
**Ireland**	County Cork	BASELINE: Babies after SCOPE	Birth cohort	Parent complete questionnaire/report	CBCL, Greenspan Social-Emotional Growth Chart
**Italy**	National	Nascita e INFanzia: gli Effetti dell’Ambiente (NINFEA)	Birth cohort	Parent complete questionnaire/report	Bespoke questionnaire; SDQ
**Netherlands**	Amsterdam	Amsterdam Born Children and their Development (ABCD)	Birth cohort	Teacher Review/Routine Education	SDQ
				Parent complete questionnaire/report	SDQ; Bespoke Questionnaire
**Netherlands**	Drenthe	GECKO Drenthe cohort (GECKO Drenthe)	Birth cohort	Parent complete questionnaire/report	SDQ (Dutch)
**Netherlands**	Rotterdam	Generation R	Birth cohort	Parent complete questionnaire/report	Bespoke (inc. IBQ-R, CBQ); ICU
**Netherlands**	Westelijke Mijnstreek region	LucKi Birth Cohort Study	Birth cohort	Parent complete questionnaire/report	Unclear
**Netherlands**	Five municipalities in the North of the Netherlands	Tracking Adolescents’ Individual Lives Survey (TRAILS) NEXT	Birth cohort	Parent complete questionnaire/report	Bespoke questionnaire
**Norway**	National	Norwegian Mother, Father and Child Cohort Study (MoBa)	Birth cohort	Parent complete questionnaire/report	CBCL; ICQ-6; EAS; SDQ; ITSEA; PPBS, RS-DBD
**Sweden**	Sweden	Child and Adolescent Twin Study in Sweden- CATSS	Birth cohort	Parent complete questionnaire/report	TCI/TCI(J); Youth Psychopathy Inventory; Child Monitoring Scale
**UK—England**	Bradford, England	Born in Bradford/Born in Bradford Growing up	Birth cohort	Parent complete questionnaire/report	SDQ
**UK—England**	Southampton	Southampton Women’s Survey	Birth cohort	Parent complete questionnaire/report	SDQ
**UK–England**	Wirral, England	Wirral Child Health and Development Study	Birth cohort	Teacher Review/Routine Education	CBCL-TRF; SDQ
				Parent complete questionnaire/report	IBQ-R; ECBQ; CBQ; BITSEA; CBCL; SDQ
**UK—National**	National	Millennium Cohort Study	Birth cohort	Parent complete questionnaire/report	SDQ
**UK—Scotland**	Scotland	Child Health Systems Programme—Pre-School (CHSP Pre-School)	Child surveillance databases	Parent complete questionnaire/report	Eyberg Child Behaviour Inventory
**UK—Scotland**	Scotland	Growing up in Scotland (GUS)	Birth cohort	Parent complete questionnaire/report	SDQ; CBQ; Pre-School Activities Inventory
**UK—Wales**	Cardiff, Wales	Cardiff Child Development Study	Birth cohort	Parent complete questionnaire/report	IBQ
**UK—Wales**	Wales	National Community Child Health Database	Child surveillance databases	Teacher Review/Routine Education	Teacher behaviour ratings

^See S3 File for abbreviations of ND measurement tools

Measurements of cognition (e.g. intelligence, attention, language, memory skills) were available in 29 data sources across 12 countries, recorded in birth cohorts, a child surveillance database, and a register of disability, see Table 3. Cognition was assessed through a varied set of approaches including clinician judgement/routine health care, direct/objective assessment, parent completed questionnaire/report and teacher review/ routine education. As well as a wide range of recognised measures such as the British Ability Scales and Weschler Intelligence Scale for Children, cognitive difficulties could also be identified through records of referral for services relating to cognitive difficulties.

**Table 3:** Data sources which record cognitive outcomes.

Country	Geographic coverage	Database name	Sub-type of data	Approach to Measurement	Measure/Scale Used^
**Denmark**	Copenhagen County	Copenhagen Child Cohort 2000 (CCC2000)	Birth cohort	Direct/Objective Assessment	WISC IV (1 subtest)
**Denmark**	Municipality of Odense	Odense Child Cohort	Birth cohort	Direct/Objective Assessment	WISC-V (4 subtest version)
**Finland**	Southwest Finland Hospital District and the Åland Islands	FinnBrain Birth Cohort Study (FinnBrain)	Birth cohort	Unclear	Unclear
**Finland**	Northern Finland	Northern Finland birth cohort of 1966	Birth cohort	Direct/Objective Assessment	WISC
**Finland**	Helsinki	Perinatal Adverse events and Special Trends in Cognitive Trajectory (PLASTICITY)	Birth cohort	Direct/Objective Assessment	ITPA, WISC, WAIS, WMS, Test of Motor Impairment, Michelsson Neurodevelopmental Screen, Benton Visual Memory Test, Goodenough Drawing Test, Frostig Test of Visual Perception, Dubowitz Developmental Screening Test, Tapping
**Finland**	10 study hospitals (Jorvi Hospital in Espoo, the Women’s Hospital and the Kätilöopisto Maternity Hospital in Helsinki, the Hyvinkää Hospital in Hyvinkää, the Kanta-Häme Central Hospital in Hämeenlinna, the Iisalmi Hospital in Iisalmi, the North Karelia Central Hospital in Joensuu, the Kuopio University Hospital in Kuopio, the Päijät-Häme Central Hospital in Lahti and the Tampere University Hospital in Tampere)	Prediction and Prevention of Preeclampsia and Intrauterine Growth Restriction (PREDO)	Birth cohort	Parent complete questionnaire/report	ASQ-III
**France**	National	Etude Longitudinale Francaise depuis l’Enfance (ELFE)	Birth cohort	Direct/Objective Assessment	BAS-II; BSRA; WISC
				Parent complete questionnaire/report	MB-CDI
**France**	Brittany	PELAGIE study (Endocrine Disruptors: Longitudinal Study on Anomalies in Pregnancy, Infertility and Childhood)	Birth cohort	Direct/Objective Assessment	WISC (subtests); Visual go/no go task
**Germany**	Munich and Nuremberg	Childhood Obesity—Early Programming by Infant Nutrition (CHOPIN)	Birth cohort	Unclear	Unclear
**Greece**	Crete	Mother Child Cohort in Crete (RHEA)	Birth cohort	Parent complete questionnaire/report	CBCL/6-18; SDQ; ADHDT
				Direct/Objective Assessment	MSCA; N-Back; Attention Network Test; Trail Making Test; Raven’s Test
**Ireland**	County Cork	BASELINE: Babies after SCOPE	Birth cohort	Direct/Objective Assessment	Kaufman Intelligence Test-II
**Italy**	eight Italian hospitals	Multiple Births Cohort Study (MUBICOS)	Birth cohort	Unclear	Unclear
**Netherlands**	Amsterdam	Amsterdam Born Children and their Development (ABCD)	Birth cohort	Direct/Objective Assessment	Amsterdam Neuropsychological Tasks
**Netherlands**	Rotterdam	Generation R	Birth cohort	Direct/Objective Assessment	NEPSY-II; BRIEF; Snijders-Oomen Non-Vernal Intelligence Test
**Netherlands**	Utrecht and its surrounding areas	YOUth Cohort study	Birth cohort	Direct/Objective Assessment	Penn word memory, Penn motor praxis test, WISC-V
**Norway**	National	Norwegian Mother, Father and Child Cohort Study (MoBa)	Birth cohort	Parent complete questionnaire/report	CDI; SLAS; CCC-2; Sprak20; EDI
**Spain**	Seven Spanish regions (Ribera d’Ebre, Menorca, Granada, Valencia, Sabadell, Asturias, and Gipuzkoa)	INMA-Environment and Childhood Project (INMA Project)	Birth cohort	Direct/Objective Assessment	MSCA; K-CPT; Batelle Developmental Inventory; California Preschool Social Competence Scale;
**Spain**	Health Area I, VI and VII of the Region of Murcia	NELA—Nutrition in Early Life and Asthma (NELA)	Birth cohort	Unclear	Unclear
**Sweden**	Sweden	Child and Adolescent Twin Study in Sweden- CATSS	Birth cohort	Direct/Objective Assessment	WISC-IV; CGAS
**Sweden**	Stockholm County	Habilitation Register	Register of disability	Clinician judgement/routine health care	Service referrals
**Sweden**	Stockholm County	Stockholm Youth Cohort	Research Cohort by Data Linkage	Clinician judgement/routine health care	ICD-10 codes
**Sweden**	Värmland county	Swedish Environmental Longitudinal, Mother and child, Asthma and allergy study	Birth cohort	Direct/Objective Assessment	Swedish Language Development Scale
**UK—England**	Avon, England	Avon Longitudinal Study of Parents & Children/Children of the 90s (ALSPAC)	Birth cohort	Direct/Objective Assessment	WPPSI; WISC; Griffiths Test
**UK—England**	Bradford, England	Born in Bradford/Born in Bradford Growing up	Birth cohort	Direct/Objective Assessment	BPVS
**UK—England**	Wirral, England	Wirral Child Health and Development Study	Birth cohort	Direct/Objective Assessment	CANTAB (IED, SWM, SOC); BPVS; WASI; BAS; Executive Function battery; Socio-Emotional Battery
**UK—National**	National	Millennium Cohort Study	Birth cohort	Direct/Objective Assessment	BAS-II, All Wales Reading Test, CANTAB (SWM/SOC)
**UK—Scotland**	Scotland	Growing up in Scotland (GUS)	Birth cohort	Direct/Objective Assessment	BAS; Children’s Communication Checklist
**UK—Wales**	Cardiff, Wales	Cardiff Child Development Study	Birth cohort	Direct/Objective Assessment	Self-regulation battery: Tower of Cardiff; Snack Delay; Whisper Task; Nonverbal Stroop card sorting test, Amsterdam Neuropsychological Tasks.
**UK—Wales**	Wales	National Community Child Health Database	Child surveillance databases	Teacher Review/Routine Education	Teacher developmental ratings

^See S3 File for abbreviations of ND measurement tools

Educational related outcomes were available in 20 data sources across 7 countries, recorded in educational databases, birth cohorts and child surveillance databases, see Table 4. All assessments were based on teacher review or routine educational data or requirement for specialist educational support. In addition to routine educational outcomes, a cohort study and a child surveillance database also had teacher Special Educational Needs ratings available.

**Table 4:** Data sources which record educational outcomes.

Country	Geographic coverage	Database name	Type of data	Approach to Measurement	Measure/Scale Used^
**Denmark**	National	Academic Achievement Register (AAR)	Educational database	Teacher Review/Routine Education	Routine Educational Outcomes
**Denmark**	National	Population’s Education Register (PER)	Educational database	Teacher Review/Routine Education	Routine Educational Outcomes
**Denmark**	National	Student Register 1	Educational database	Teacher Review/Routine Education	Routine Educational Outcomes
**Denmark**	National	Student Register 2	Educational database	Teacher Review/Routine Education	Routine Educational Outcomes
**Finland**	National	Discontinuation of education	Educational database	Teacher Review/Routine Education	Routine Educational Outcomes
**Finland**	Helsinki	Perinatal Adverse events and Special Trends in Cognitive Trajectory (PLASTICITY)	Birth cohort	Teacher Review/Routine Education	Routine Educational Outcomes
**Finland**	National	Upper secondary general school education	Educational database	Teacher Review/Routine Education	Routine Educational Outcomes
**Italy**	National	Nascita e INFanzia: gli Effetti dell’Ambiente (NINFEA)	Birth cohort	Teacher Review/Routine Education	Parent reported grades
**Netherlands**	Amsterdam	Amsterdam Born Children and their Development (ABCD)	Birth cohort	Teacher Review/Routine Education	CITO Index
**Norway**	National	National Education Database NUDB.	Educational database	Teacher Review/Routine Education	Routine educational outcomes
**Sweden**	National	The Swedish Register of Education	Educational database	Teacher Review/Routine Education	Routine education outcomes
**UK—England**	Avon, England	Avon Longitudinal Study of Parents & Children/Children of the 90s (ALSPAC)	Birth cohort	Teacher Review/Routine Education	Routine education outcomes; teacher rated questionnaires
**UK—England**	Bradford, England	Born in Bradford/Born in Bradford Growing up	Birth cohort	Teacher Review/Routine Education	Routine education outcomes; local authority data
**UK—England**	England	Community Services Data Set (CSDS)	Child surveillance databases	Teacher Review/Routine Education	Teacher Special Educational Need (SEN) ratings
**UK—England**	Two acute and one Mental Health Care National Health Service (NHS) Provider in South London	Early Life Cross Linkage in Research (eLIXIR) Partnership	Research Cohort by Data Linkage	Teacher Review/Routine Education	Routine education outcomes
**UK—England**	England	National Pupil Database	Educational database	Teacher Review/Routine Education	Routine education outcomes
**UK—National**	National	Millennium Cohort Study	Birth cohort	Teacher Review/Routine Education	Teacher Special Educational Need (SEN) ratings; Routine education outcomes
**UK—Scotland**	Scotland	Achievement of Curriculum for Excellence Levels	Educational database	Teacher Review/Routine Education	Routine education outcomes
**UK—Wales**	Wales	Education Attainment	Educational database	Teacher Review/Routine Education	Routine education outcomes
**UK—Wales**	Wales	National Community Child Health Database	Child surveillance databases	Teacher Review/Routine Education	Teacher SEN ratings

^See S3 File for abbreviations of ND measurement tools

Neurodevelopmental disorder diagnostic codes were available in 42 data sources across 11 countries, see Table 5. The presence of ND disorders was based on parent completed questionnaire/report, direct/objective assessment, child self-report, clinician judgement/routine health care, teacher review/routine education and direct/objective assessment. Diagnoses recorded using ICD-9, ICD-10, ICPC (International Classification for Primary Care), DSM (Diagnostic and Statistical Manual of Mental Disorders) IV, and Read codes were available in all database types except for education. ND measures such as the Checklist for autism in toddlers (CHAT), Childhood Asperger Syndrome Test (CAST) and Attention Deficit/Hyperactivity Disorder Test (ADHD) to identify children with autism, Asperger syndrome or ADHD were exclusively found in birth cohorts, although one child surveillance database had results for the Modified Checklist for Autism in Toddlers (M-CHAT).

**Table 5:** Data sources which record neurodevelopmental disorders.

Country	Geographic coverage	Database name	Sub-type of data	Approach to Measurement	Measure/Scale Used^
**Denmark**	National	ADHD Database	Disease registry	Clinician judgement/routine health care	ICD codes
**Denmark**	National (Denmark, Greenland and the Faroes)	Central Psychiatric Register	Hospital databases*	Clinician judgement/routine health care	ICD Codes
**Denmark**	Copenhagen County	Copenhagen Child Cohort 2000 (CCC2000)	Cohort study	Parent complete questionnaire/report	CHAT, DAWBA
				Clinician judgement/routine health care	ICD-10, DSM IV codes
**Denmark**	National	National Patient Register	Hospital databases*	Clinician judgement/routine health care	ICD codes
**Denmark**	Municipality of Odense	Odense Child Cohort	Cohort study	Parent complete questionnaire/report	SRS; ADHD-Rating Scale
**Finland**	National	Care Register for Health Care (HILMO) (replaced the Hospital Discharge Register in 1994)	Hospital databases*	Clinician judgement/routine health care	ICD-9; ICD-10 in recent years
**Finland**	Northern Finland	Northern Finland birth cohort of 1966	Cohort study	Direct/Objective Assessment	WISC
**Finland**	National	Primary health care (AvoHILMO)	Primary care database	Clinician judgement/routine health care	ICD-10/ICPC codes
**France**	National	French national health data system (SNDS), health insurance claim and hospital discharge databases	Hospital database	Clinician judgement/routine health care	ICD-10 codes
**Germany**	Munich and Nuremberg	Childhood Obesity—Early Programming by Infant Nutrition (CHOPIN)	Cohort study	Unclear	Unclear
**Germany**	17% of national population	German Pharmacoepidemiological Research Database (GePaRD): Hospital data and Outpatient data	Administrative health insurance claims database	Clinician judgement/routine health care	ICD-10 codes
**Ireland**	National	National Ability Supports System (created in 2018 by merging National Intellectual Disability Database (NIID) and National Physical and Sensory Disability Database (NPSDD))	Register of disability	Clinician judgement/routine health care	ICD-10; service use records
**Italy**	Florence, Rome, Trieste, Turin and Viareggio	Piccolipiù	Cohort study	Unclear	Unclear
**Italy**	Emilia Romagna Region	SINPIA ER-Flusso informativo per i servizi di neuropsichiatria infantile dell’infanzia e dell’adolescenza dell’Emilia Romagna	Hospital databases*	Clinician judgement/routine health care	ICD-10; service use records
**Italy**	Tuscany	Tuscany SALM–mental health services	Hospital databases*	Clinician judgement/routine health care	ICD-10
**Netherlands**	Rotterdam	Generation R	Cohort study	Child Self-Report	AQ-Short
				Direct/Objective Assessment	Autism Diagnostic Interview—Revised
				Parent complete questionnaire/report	SRS
**Netherlands**	25% of the Netherlands	PHARMO-PRN cohorts	Research Cohort by Data Linkage	Clinician judgement/routine health care	ICD codes
**Netherlands**	National	PRIDE Study (PRIDE: PRegnancy and Infant DEvelopment)	Cohort study	Clinician judgement/routine health care	Routine medical records
**Norway**	National	Norwegian Mother, Father and Child Cohort Study (MoBa)	Cohort study	Parent complete questionnaire/report	ESAT; M-CHAT; CAST; CPRS-R
**Norway**	National	Norwegian Patient Registry (NPR)	Hospital databases*	Clinician judgement/routine health care	ICD-10 codes
**Norway**	National	Norwegian Registry for Primary Health Care	Primary care database	Clinician judgement/routine health care	ICD-10/ICPC codes
**Spain**	Catalonia	Information system for research in primary care (SIDIAP)	Primary care database	Clinician judgement/routine health care	ICD-10
**Spain**	Seven Spanish regions (Ribera d’Ebre, Menorca, Granada, Valencia, Sabadell, Asturias, and Gipuzkoa)	INMA-Environment and Childhood Project (INMA Project)	Cohort study	Clinician judgement/routine health care	ADHD Criteria of DSM-IV; CAST
**Spain**	Seven Spanish regions (Ribera d’Ebre, Menorca, Granada, Valencia, Sabadell, Asturias, and Gipuzkoa)	INMA-Environment and Childhood Project (INMA Project)	Cohort study	Parent complete questionnaire/report	ADHD Criteria of DSM-IV; CAST
**Sweden**	South East	All Babies in Southeast Sweden (ABIS)	Cohort study	Clinician judgement/routine health care	ICD-9/ICD-10 codes
**Sweden**	Sweden	Child and Adolescent Twin Study in Sweden- CATSS	Cohort study	Child Self-Report	ADHD self-report scale
				Clinician judgement/routine health care	A-TAC; ASDI
				Parent complete questionnaire/report	CBCL
**Sweden**	Stockholm County	Clinical Database for Child and Adolescent Psychiatry in Stockholm	Hospital databases*	Clinician judgement/routine health care	DSM-IV/ICD-10 codes
**Sweden**	National	National Patient Register	Hospital databases*	Clinician judgement/routine health care	ICD-0 codes
**Sweden**	Stockholm County	Stockholm Adult Psychiatric Care Register	Hospital databases*	Clinician judgement/routine health care	DSM-IV/ICD-10 codes
**Sweden**	Stockholm County	Stockholm Youth Cohort	Research Cohort by Data Linkage	Clinician judgement/routine health care	DSM-IV/ICD-10 codes; Service referrals
**Sweden**	Stockholm County	VAL database	Hospital databases*	Clinician judgement/routine health care	ICD-10 codes; referrals
**UK—England**	Two acute and one Mental Health Care National Health Service (NHS) Provider in South London	Early Life Cross Linkage in Research (eLIXIR) Partnership	Research Cohort by Data Linkage	Clinician judgement/routine health care	Read codes
**UK—England**	England	ResearchOne database	Research Cohort by Data Linkage	Clinician judgement/routine health care	ICD-10 codes; referrals (Read codes)
**UK—England**	Wirral, England	Wirral Child Health and Development Study	Cohort study	Parent complete questionnaire/report	DAWBA; Connors Checklist; SCQ
**UK—National**	National	Clinical Practice Research Datalink	Primary care database	Clinician judgement/routine health care	ICD-10 codes, referrals (Read codes
**UK—National**	National	The Health Improvement Network (THIN)	Primary care database	Clinician judgement/routine health care	Read codes; referrals
**UK—Northern Ireland**	Northern Ireland	General Practitioner Information Platform	Primary care database	Clinician judgement/routine health care	Read codes
**UK—Scotland**	Scotland	Child Health Systems Programme—Pre-School (CHSP Pre-School)	Child surveillance databases	Parent complete questionnaire/report	M-CHAT
**UK—Scotland**	Scotland	Child Health Systems Programme—School (CHSP School)	Child surveillance databases	Clinician judgement/routine health care	ICD-10 codes, referrals
**UK—Scotland**	Scotland	Support Needs System (SNS)	Register of disability	Clinician judgement/routine health care	Referrals
**UK—Wales**	Cardiff, Wales	Cardiff Child Development Study	Cohort study	Parent complete questionnaire/report	CBCL/1.5–5; Developmental Milestones Questionnaire; Connors 3 ADHD Index-Parent Report
				Teacher Review/Routine Education	CBCL-TRF
				Direct/Objective Assessment	Preschool Age Psychiatric Assessment
**UK—Wales**	70% of Wales	Primary Care GP dataset	Primary care database	Clinician judgement/routine health care	ICD- 10 codes
**UK—Wales, Scotland, Northern Ireland**	Wales, Scotland, Northern Ireland	The National Neonatal Research database (NNRD)	Disease registry	Clinician judgement/routine health care	Neurological diagnoses

* Admission, Episode, Discharge

^See S3 File for abbreviations of ND measurement tools

## Discussion

Pharmacoepidemiologic investigations into ND outcomes in children exposed to a medication during pregnancy lag behind initiatives to understand risk of congenital anomaly. We identified 90 data sources, across 14 countries, which contain information on five domains of ND–infant development, child behaviour, cognitive, education and ND disorders. It is hoped that this inventory of potentially linkable data sources will expediate investigations into risk of ND outcomes in children associated with medication exposures in pregnancy. When selecting data sources for such research it is important to consider the ND outcome reported, the sensitivity and specificity of measurement, variability in measurement approach and the trajectory of skill development. For certain ND outcomes there is continued progress into the second decade of life [27], due to continued development of the architecture in regions of the brain [28]. Each of these is discussed in more detail below.

Although we grouped the ND outcomes available in the data sources into five domains, the measure/scale used, including the presence or absence of medical diagnoses, results of psychometric instruments (questionnaires or tests) completed by parents, teachers, or health care professionals, educational assessments, and registration of children in disability registers and how these were recorded, these varied within each domain. With such variation, the groups are intended to be informative, highlighting data which could be available for that ND outcome. It does not mean that data are collected in a similar enough manner across data sources to be combined directly in analysis. For example, within the cognitive domain there were continually measured IQ scores as well as ICD-10 and other diagnostic codes relating to intellectual disability. The former was measured with a variety of different measures on a continuous scale, yet the latter represents a diagnosis which is likely to only refer to the most severe cases of intellectual difficulties [29, 30]. The suggested groups should be used in future initiatives to direct researchers to data sets that may be available, but mapping exercises, with expert input, will be required to understand the comparability of the data available within each of the data sources for specific research questions.

ND outcomes recorded in birth cohorts and child surveillance databases were more likely to be based on psychometric tests performed on all children included in the cohort. ND outcomes recorded in primary care databases, hospital databases and administrative health insurance claims databases were nearly always based on diagnostic codes. Frequently, ND outcomes based on psychometric instruments were recorded on a continuous scale, increasing the sensitivity and specificity of the outcome data collected, as the functioning of the entire cohort is available. Higher measurement sensitivity reduces the required cohort size and therefore smaller sized cohorts with these measurements can be useful sources. When using diagnostic codes as a marker of the presence or absence of a diagnosis (i.e., ASD or ADHD) it must be recognised that these are based on routine care practices, which only capture the most affected individuals and are only truly accurate in those who were formally reviewed for the diagnosis. The level of social communication and interaction ability in those without the diagnostic code for autism for example is unknown and there is clear evidence that diagnostic processes are influenced by family background, ethnicity, parental education and socioeconomic status [31, 32]. Thus, children may experience moderate levels of disruption of function but may not either reach diagnostic thresholds or never be reviewed for the diagnostic code in question. Therefore, different data sources may be utilised in different ways, to answer different questions and both will have their inherent strengths and limitations about the sensitivity and specificity of the measurement of the ND outcome.

Both the approaches to measuring ND outcomes include variability in assessment of ND outcomes. The birth cohorts and child surveillance datasets included a broad selection of standardised psychometric instruments e.g., Bayley Scales of Infant Development to assess early development, or Wechsler Preschool and Primary Scale of Intelligence to assess child IQ. National diversity in healthcare provision and practice also contributes to variability when combining diagnostic data across healthcare systems and countries due to variability in regional and national approaches to diagnosis [33]. It should also be recognised that the diagnostic criteria for certain disorders has varied over time and across countries. Variability needs to be considered by the users of the data sources and steps taken to maximise the comparability of data. For some data sources such as the primary care databases, which do not tend to be standardised, this may include standardisation and validation of the data before they can be used. This increases the time and cost of using the data and requires collaboration with local data providers and experts [34].

The heterogeneity is even more pronounced when it comes to educational outcomes, where educational systems and teacher’s assessment are country specific. One way to report a common indicator is to assess the proportion of children above or below the average or the proportion of children considered to pass a specific routine exam. However, this proposal is not without difficulties, as children sit formal examinations at different ages across Europe. For instance, in the UK, children are tested at 4 key stages (ages 5–7, 8–11, 12–14, and 15–16). In contrast, children in Finland sit their first examinations at age 16 years. Educational data and child health surveillance data have been used much less frequently to determine ND risk than other types of data. However, they have the benefit that data are available for the whole population, not just those referred with a suspected diagnosis, and represent some domains of ND over the long-term/teenage years.

Finally, it should be considered that all data sources have potential for bias. In countries or regions with health registries, it may be comparatively cheap and fast to use diagnosis codes. However, detection bias cannot be ruled out. For example, it is not possible to blind a child’s exposure status from the health care professionals reviewing the child to rate the ND outcome. Whilst, this may be less of an influence early on, once an association between a medication exposure and a child ND outcome has been established this may positively bias practice. For example, only prescribing this medication to clinically severe patients whose disease cannot be controlled with less teratogenic or toxic medications. Additional biases come from population health behaviour. For instance, it is suspected that women exposed to a suspected teratogen, or women with a history of mental illness, may be more likely to get health or developmental referrals for their children.

### Strengths and weaknesses

It is not possible to confirm that every data source has been identified and is included in the inventory. This is particularly true for databases that are not included in published papers, databases that are used for medications other than SSRIs or in European countries with no EUROCAT/EUROmediCAT or Euro-Peristat contact available. However, given the global move towards using electronic administrative databases for research, it is likely that we have identified the databases that are most accessible for pharmacoepidemiologic research.

A pre-requisite for inclusion was that a data source should contain information on maternal medication use in pregnancy, either in the data source itself or through linkage with another data source. The level of detail relating to maternal medication use will vary, potentially impacting how useful these data sources are when examining the impact of medication use in pregnancy on ND outcomes. Data sources which can be linked to, for example, large administrative prescribing databases could potentially provide quite detailed information such as specific drug name/code, dates of prescribing/dispensation, dose, and route of administration. However, maternal use of over-the-counter medication would not be available. In contrast, in birth cohorts, where the impact of maternal medication use in pregnancy is not the primary research question, limited maternal medication use may be recorded. For example, exposure to broad drug groups such as antibiotics or anticonvulsants may be recorded rather than the specific drug. However, over the counter medication use may be available.

Data sources were not contacted to confirm if they would allow their data i.e. medication exposure records or ND outcome data to be used in secondary research. In a similar piece of work the response rate was just 52% from data sources contacted [35]. Where data sources, in particular birth cohorts, do not have websites or publicly available documentation relating to methodology the determination of ND measures available relied on published articles. It may be possible that such data sources hold more data relating to ND outcomes than have been identified. For six birth cohorts it was not possible to determine the methods by which ND was assessed (1 assessing infant development, 1 child behaviour, 4 cognitive and 2 neurodevelopmental disorder assessments).

## Conclusions

Ninety European data sources were identified with potential to be used to assess five domains of ND following maternal medication use during pregnancy. These have great potential to be used in pharmacoepidemiologic research into the safety of SSRIs and other medications in pregnancy potentially associated with ND outcomes in children. Caution must be used when combining varied approaches to ND outcome measurement and consideration regarding the sensitivity and specificity of the outcome measure selected and the age of the child at review/follow up should be borne in mind. This inventory is an invaluable resource for researchers planning future studies to investigate the ND impact of medication exposures during pregnancy.

## Supporting information

S1 File Search query. (DOCX)

S2 File Types of data source with ND outcomes available. (DOCX)

S3 File Abbreviations of ND measurement tools. (DOCX)
