# Supplementary material for: An inventory of European data sources to support pharmacoepidemiologic research on neurodevelopmental outcomes in children following medication exposure in pregnancy: A contribution from the ConcePTION project
Source: PLoS One. 2022 Oct 14;17(10):e0275979. doi: 10.1371/journal.pone.0275979 (PMC9565459; doi:10.1371/journal.pone.0275979)
Supplement: S1 File — (DOCX) [file pone.0275979.s001.docx]

## S1 Supplementary file 1 Search Query

SEARCH QUERY,"(('pregnancy'/exp OR 'lactation'/exp) AND ('depression'/exp OR 'serotonin uptake inhibitor'/exp OR 'serotonin noradrenalin reuptake inhibitor') AND ('juvenile'/exp AND ('perinatal outcome'/exp OR 'low birth weight'/exp OR 'congenital malformation'/exp OR 'mental disease'/exp))) AND (2009:py OR 2010:py OR 2011:py OR 2012:py OR 2013:py OR 2014:py OR 2015:py OR 2016:py OR 2017:py OR 2018:py OR 2019:py) AND ('case control study'/de OR 'cohort analysis'/de OR 'controlled study'/de OR 'cross-sectional study'/de OR 'drug surveillance program'/de OR 'evidence based practice'/de OR 'human'/de OR 'longitudinal study'/de OR 'meta-analysis'/de OR 'observational study'/de OR 'population based case control study'/de OR 'prospective study'/de OR 'retrospective study'/de OR 'systematic review'/de) AND ('article'/it OR 'article in press'/it OR 'review'/it)"
