# Supplementary material for: An inventory of European data sources to support pharmacoepidemiologic research on neurodevelopmental outcomes in children following medication exposure in pregnancy: A contribution from the ConcePTION project
Source: PLoS One. 2022 Oct 14;17(10):e0275979. doi: 10.1371/journal.pone.0275979 (PMC9565459; doi:10.1371/journal.pone.0275979)
Supplement: S2 File — (DOCX) [file pone.0275979.s002.docx]

## S2 Supplementary file 2 Types of data source with ND outcomes available

**Healthcare databases** – created for operational health care purposes and reimbursement of costs. Include:

- **Hospitalisation (Admission/Episode/Discharge) databases** - for inpatient/outpatient care. Include Maternity databases (for care given to pregnant women up to delivery) and specialist databases such as Neonatal Intensive Care, fetal medicine and paediatric surgery databases.
- **Primary care databases** – healthcare provided by General Practitioners (GPs). Includes prescriptions issued.
- **Administrative health insurance claims database** – for reimbursement of healthcare costs. May be specific to a single insurer or combined across insurance schemes. May include both primary and hospital care.
- **Child surveillance databases** – record growth and development as measured by community child health teams/public health nurses.

**Other administrative databases** – other operational databases for the delivery of services, reimbursement of costs.

- **Educational databases** - created for operational education administration purposes for example school results, special educational needs, and attendance.
- **Register of disability** - created for insurance purposes and service delivery.

**Health surveillance and research databases** – created for public health surveillance or research. May be derived from healthcare databases with extra validation or notification by clinicians or based on special recruitment and patient interview. Include:

- **Disease registries** - such as Congenital anomaly, Cancer, Cerebral Palsy or Diabetes registries.
- **Birth cohorts** - recruit pregnant women during pregnancy or at birth, irrespective of exposure, and follow-up prospectively.
- **Research Cohort by Data Linkage** – linkage of several healthcare or administrative data sources so that they can be used to conduct research into a specific area. An example would be EFEMERIS which can be used to monitor the prescription of reimbursed drugs to French pregnant women as well as to identify adverse pregnancy outcomes such as congenital malformations or effects on psychomotor development of the child.

*Excluded*
**Prospective Study Database** – created for the purposes of a distinct research project such as a Randomised Controlled Trial. Often limited in size.

**Adverse drug reaction databases** – databases notified of adverse health outcomes associated with medication exposure.
