# Supplementary material for: An inventory of European data sources to support pharmacoepidemiologic research on neurodevelopmental outcomes in children following medication exposure in pregnancy: A contribution from the ConcePTION project
Source: PLoS One. 2022 Oct 14;17(10):e0275979. doi: 10.1371/journal.pone.0275979 (PMC9565459; doi:10.1371/journal.pone.0275979)
Supplement: S3 File — (DOCX) [file pone.0275979.s003.docx]

## S3 Supplementary file 3 Abbreviations of ND measurement tools

| **Abbreviation** | **ND measurement tool** | **Assessment method** |
| --- | --- | --- |
| ADHDT | Attention Deficit/Hyperactivity Disorder Test | Questionnaire |
| ANT | Amsterdam Neuropsychological Tasks. | Direct Assessment/Diagnostic Interview |
| AQ-Short | Autism-Spectrum Quotient Test | Questionnaire |
| ASDI | Asperger Syndrome Diagnostic Interview | Direct Assessment/Diagnostic Interview |
| ASQ | Ages and stages questionnaire | Questionnaire |
| A-TAC | The Autism–Tics, ADHD and other Comorbidities inventory | Direct Assessment/Diagnostic Interview |
| BAS | British Ability Scales | Direct Assessment/Diagnostic Interview |
| BITSEA | Brief Infant-Toddler Social and Emotional Assessment | Questionnaire |
| BITSEA | Brief Infant-Toddler Social and Emotional Assessment | Questionnaire |
| BPVS | British Picture Vocabulary Scale | Direct Assessment/Diagnostic Interview |
| BPVS | British Picture Vocabulary Scale | Direct Assessment/Diagnostic Interview |
| BRIEF | Behavior Rating Inventory of Executive Function | Questionnaire |
| BS | Barkely Scales | Questionnaire |
| BSID | Bayley Scales of Infant Development | Direct Assessment/Diagnostic Interview |
| BSRA | Bracken School Readiness Assessment | Questionnaire |
| CANTAB | Cambridge Cognition | Direct Assessment/Diagnostic Interview |
| CANTAB - IED | Cambridge Cognition - Intra-Extra Dimensional Set Shift | Direct Assessment/Diagnostic Interview |
| CANTAB - SOC | Cambridge Cognition - Stockings of Cambridge | Direct Assessment/Diagnostic Interview |
| CANTAB - SWM | Cambridge Cognition - Spatial Working Memory | Direct Assessment/Diagnostic Interview |
| CAST | Childhood Asperger Syndrome Test | Questionnaire |
| CBCL/1.5-5 | Child Behavior Checklist - 1.5 to 5 years | Questionnaire |
| CBCL/6-18 | Child Behavior Checklist - 6 to 18 years | Questionnaire |
| CBCL-TRF | CBCL - Teacher Report Form | Questionnaire |
| CBCL-YSR | CBCL - Youth Self Report | Questionnaire |
| CBQ | Child Behavior Questionnaire | Questionnaire |
| CCC-2 | Children's Communication Checklist | Questionnaire |
| CDI | Child Development Inventory | Questionnaire |
| CGAS | Children's Global Assessment Scale | Questionnaire |
| CHAT | Checklist for Autism in Toddlers | Questionnaire |
| CITO Index | Central Institute for Test Development | Questionnaire |
| CKAT | Clinical Kinematic Assessment Tool | Direct Assessment/Diagnostic Interview |
| CPRS-R | Conners Parent Rating Scales - Revised | Questionnaire |
| DAWBA | Development & Well-Being Assessment | Questionnaire |
| DBD-RS | Disruptive Behaviour Disorder Rating Scale | Questionnaire |
| EAS | Emotional Activity & Sociability Questionnaire | Questionnaire |
| ECBQ | Early Childhood Behavior Questionnaire | Questionnaire |
| ECBQ-R | Early Childhood Behavior Questionnaire - Revised | Questionnaire |
| EDI | Early Development Instrument | Questionnaire |
| ESAT | Early Screening of Autistic Traits | Questionnaire |
| GMFCS | Gross Motor Function Classification System | Direct Assessment/Diagnostic Interview |
| IBQ | Infant Behavior Questionnaire | Questionnaire |
| IBQ-R | Infant Behavior Questionnaire-Revised | Questionnaire |
| ICQ-6 | Infant Characteristics Questionnaire - 6 months | Questionnaire |
| ICU | Inventory of Callous-Unemotional Traits | Questionnaire |
| ITPA | Illinois Test of Psycholinguistic Abilities | Direct Assessment/Diagnostic Interview |
| ITSCL | Infant Toddler Symptom Checklist | Questionnaire |
| ITSEA | Infant and Toddler Social and Emotional Assessment | Questionnaire |
| K-CPT | Conners' Kiddie Continuous Performance Test | Direct Assessment/Diagnostic Interview |
| Lab-TAB | Laboratory Temperament Assessment Battery | Direct Assessment/Diagnostic Interview |
| MB-CDI | MacArthur-Bates Communicative Development Inventory | Direct Assessment/Diagnostic Interview |
| M-CHAT | Modified Checklist for Autism in Toddlers | Questionnaire |
| MSCA | McCarthy Scales of Child Abilities | Direct Assessment/Diagnostic Interview |
| NBAS | Neonatal British Ability Scales | Direct Assessment/Diagnostic Interview |
| NEPSY | A Developmental NEuroPSYchological Assessment | Direct Assessment/Diagnostic Interview |
| NVCC | Non-Verbal Communication Checklist | Questionnaire |
| PEDS | Parents' Evaluation of Developmental Status | Questionnaire |
| PEDS(DM) | Parents' Evaluation of Developmental Status (Developmental Milestones) | Questionnaire |
| PPBS | Pre-school play behavior scale | Questionnaire |
| RS-DBD | Rating Scale for Disruptive Behavior Disorders | Questionnaire |
| SCPE | Surveillance of Cerebral Palsy in Europe | Direct Assessment/Diagnostic Interview |
| SCQ | Social Communication Questionnaire | Questionnaire |
| SDQ | Strengths & Difficulties Questionnaire | Questionnaire |
| SGS | Schedule of Growing Skills | Direct Assessment/Diagnostic Interview |
| SLAS | Speech and Language Assessment Scale | Questionnaire |
| SOGS II | Schedule of Growing Skills II | Direct Assessment/Diagnostic Interview |
| SON-R | Snijders-Oomen Non-Verbal Intelligence Test | Direct Assessment/Diagnostic Interview |
| Sprak20 | Checklist for Language Related Difficulties | Questionnaire |
| SRS | Social Responsiveness Scale | Questionnaire |
| SSLM | Sure Start Language Measure | Questionnaire |
| TCI (J) | Temperament & Character Inventory (Junior) | Questionnaire |
| WAIS | Weschler Adult Intelligence Scale | Direct Assessment/Diagnostic Interview |
| WASI | Weschler Abbreviated Scale of Intelligence | Direct Assessment/Diagnostic Interview |
| WISC | Weschler Intelligence Scale for Children | Direct Assessment/Diagnostic Interview |
| WMS | Weschler Memory Scale | Direct Assessment/Diagnostic Interview |
| WPPSI | Wechsler Preschool and Primary Scale of Intelligence | Questionnaire |
| YSR | Youth Self-Report | Questionnaire |
