# Supplementary material for: An inventory of European data sources to support pharmacoepidemiologic research on neurodevelopmental outcomes in children following medication exposure in pregnancy: A contribution from the ConcePTION project
Source: PLoS One. 2022 Oct 14;17(10):e0275979. doi: 10.1371/journal.pone.0275979 (PMC9565459; doi:10.1371/journal.pone.0275979)
Supplement: Attachment — Submitted filename: Reviews.docx [file pone.0275979.s004.docx]

1. The aim of this paper needs to be justified, given the project has been established.

2. The age of children would be important for the study of neurodevelopmental disorders. Thus, it would be very informative to list the start (end) year of the cohorts and registers to identify the children.

3. L371-379: The authors stated that detection bias is always possible when using health registries. To my knowledge, detection bias is less likely in registers since the prescribers and clinicians who see the children are often different. Moreover, as the author mentioned, a large proportion of women take medications during pregnancy. Therefore, medications do not necessarily influence the doctors’ diagnosis. For instance, clinicians may be more reluctant to prescribe the medication to pregnant women unless they have severe disorders when an association is established. It is difficult to know whether any associations are due to positively biased practice or indication for treatment.

4. There are still cohorts/registers missing from the ConcePTION project. Please clarify whether this project will be static, i.e., restricting to these 90 data sources, or be dynamic by identifying more relevant resources?
